# Antibiotic use and consumption among medical patients of two hospitals in Sierra Leone: a descriptive report

**DOI:** 10.1186/s12879-023-08517-0

**Published:** 2023-10-27

**Authors:** Sulaiman Lakoh, Christine Ellen Elleanor Williams, Stephen Sevalie, James B.W. Russell, Sarah K. Conteh, Joseph Sam Kanu, Umu Barrie, Gibrilla F. Deen, Anna Maruta, Daniel Sesay, Olukemi Adekanmbi, Darlinda F. Jiba, Joseph Chukwudi Okeibunor, George A. Yendewa, Emmanuel Firima

**Affiliations:** 1 College of Medicine and Allied Health Sciences, University of Sierra Leone, New England, Freetown, Sierra Leone; 2 Ministry of Health and Sanitation, Government of Sierra Leone, Freetown, Sierra Leone; 3 Sustainable Health Systems Sierra Leone, Freetown, Sierra Leone; 4 Infectious Disease Research Network, Freetown, Sierra Leone; 5 34 Military Hospital, Freetown, Sierra Leone; 6 World Health Organization Country Office, Freetown, Sierra Leone; 7 Department of Medicine, College of Medicine, University of Ibadan, Ibadan, Nigeria; 8 Department of Medicine, University College Hospital, Ibadan, Nigeria; 9 World Health Organization Regional Office for Africa, Brazzaville, Congo; 10 Department of Medicine, Case Western Reserve University School of Medicine, Ohio, USA; 11 Division of Infectious Diseases and HIV Medicine, University Hospitals Cleveland Medical Center, OH Cleveland, USA; 12 Johns Hopkins Bloomberg School of Public Health, MD Baltimore, USA; 13 Department of Medicine, Clinical Research Unit, Swiss Tropical and Public Health Institute, Basel, Switzerland; 14 University of Basel, Basel, Switzerland; 15 SolidarMed, Maseru, Lesotho; 16 Centre for Multidisciplinary Research and Innovation, Abuja, Nigeria

**Keywords:** Defined daily dose (DDD), ACCESS, WATCH and RESERVE (AWaRe), Antibiotic consumption, Sierra Leone, Antimicrobial resistance (AMR)

## Abstract

**Background:**

Although one of the main drivers of antimicrobial resistance is inappropriate antibiotic prescribing, there are limited resources to support the surveillance of antibiotic consumption in low-income countries. In this study, we aimed to assess antibiotic use and consumption among medical patients of two hospitals in different geographic regions of Sierra Leone.

**Methods:**

This is a cross-sectional study of adult (18 years or older) patients receiving medical care at two hospitals (34 Military Hospital-MH and Makeni Government Hospital-MGH) between March 2021 and October 2021. After admission to the medical or intensive care unit, patients were sequentially recruited by a nurse from each hospital. Demographic and clinical characteristics and information on the dose of antibiotics, their routes, and frequency of administration and duration were collected using a questionnaire adapted from previous studies and encrypted in EpiCollect software (Epic, Verona WI). A physician reviews and verifies each completed questionnaire. Data analysis was done using STATA version 16.

**Results:**

The mean age of the 468 patients evaluated in this study was 48.6 years. The majority were women (241, 51.7%) and treated at MGH (245, 52.0%). Clinical diagnosis of bacterial infection was made in only 180 (38.5%) patients. Regardless of the diagnosis, most (442, 94.9%) patients received at least one antibiotic. Of a total 813 doses of antibiotics prescribed by the two hospitals, 424 (52.2%) were administered in MH. Overall, antibiotic consumption was 66.9 defined daily doses (DDDs) per 100 bed-days, with ceftriaxone being the most commonly used antibiotic (277, 34.1%). The ACCESS and WATCH antibiotics accounted for 18.9 DDDs per 100 bed-days (28.2%) and 48.0 DDDs per 100 bed-days (71.7%), respectively. None of the patients were prescribed a RESERVE antibiotics. The antibiotic consumption was lower in MH (61.3 DDDs per 100 bed-days) than MGH (76.5 DDDs per 100 bed-days).

**Conclusion:**

Antibiotic consumption was highest with ceftriaxone, followed by levofloxacin and metronidazole. Given the high rate of consumption of antibiotics in the WATCH category of the AWaRe classification, there is a need to initiate surveillance of antibiotic consumption and establish hospital-based antibiotic stewardship in these settings.

## Introduction

Owing to its increasing burden, antimicrobial resistance (AMR) is a major threat to the health, social and economic status of countries around the world [1]. The global death toll from AMR was estimated at 700,000 in 2014 [1]. In a recent estimate, bacterial AMR alone was responsible for 1.27 million global deaths in 2019, exceeding the number of deaths caused by malaria and HIV over the same period [1, 2]. This fact demonstrates the trajectory of the increasing burden of AMR and supports previous estimates that if left unchecked, AMR will cause 10 million deaths per year globally by 2050 [1].

AMR is a complex phenomenon that affects human health in community and hospital settings and increases healthcare expenditures, prolongs hospital stays, and worsens morbidity and mortality [3]. One of the main drivers of AMR in both the community and hospital settings is the high rates of inappropriate antibiotic prescriptions reported by many studies in low- and middle-income countries (LMICs) [4–6]. For example, increased antibiotic consumption in LMICs has driven a nearly 40% increase in global antibiotic consumption over the past 30 years [7].

Even with this development, many LMICs have limited capacity to initiate antimicrobial consumption surveillance and implement antimicrobial stewardship (AMS) activities needed to reduce inappropriate antibiotic consumption [8–10]. The COVID-19 pandemic has created additional challenges as AMS-related activities were de-prioritized at the peak of the pandemic [11, 12]. Therefore, it is necessary to optimize AMS activities to protect essential antibiotics and prevent the development of AMR in public health emergencies [12]. One strategy is to use standardized international classification systems to assess consumption and use patterns. The World Health Organization (WHO) established the ACCESS, WATCH, and RESERVE (AWaRe) Framework, the Anatomical Therapeutic Chemical (ATC) Classification system, and the Defined Daily Dose (DDD) per 100 hospital bed-days as standardized classification tools to evaluate antibiotic use patterns and monitor antimicrobial consumption [13–16].

Despite these resources and the fact that previous observational studies in Sierra Leone have identified ingrained challenges in the response against AMR [17–19], there are few studies assessing antibiotic consumption in Sierra Leone. A March 2022 PubMed search revealed only three articles on antibiotic use and/or consumption in Sierra Leone, none of which focused specifically on assessing antibiotic use and consumption among medical patients [6, 20, 21].

Hence, this study set out to (a) determine the prevalence antibiotic use among patients admitted in medical wards of two hospitals in Sierra Leone (b) evaluate antibiotic consumption using the 2021 ATC/DDD classification, and (c) identify patterns of antibiotic consumption using the AWaRe framework.

## Methods and materials

### Study design

The study used a cross-sectional design to assess antibiotic use and consumption among patients admitted in the medical wards of two hospitals in Sierra Leone.

### Study population and study duration

The study included all adult inpatients aged 18 years or older admitted to the Medical Wards and intensive care units (ICU) of two hospitals between March 2021 and October 2021.

### Study setting

We selected 34 Military Hospital (MH) and Makeni Government Hospital (MGH) for the study as they are in two different geographic regions and their level of service provision and infrastructure is likely representative of many secondary or tertiary hospitals in Sierra Leone. Both hospitals provide tertiary services. MH is located in Freetown, the capital of Sierra Leone, with a population of one million and 181 beds. MGH is a regional hospital, located approximately 170 km from Freetown, has a catchment population of 606,544 people (approximately 8.6% of Sierra Leone’s population) and 207 beds [22]. Approximately 33% (60) of the beds in MH and 22% (44) of the beds in MGH are in the medical wards.

The two hospitals have laboratories with capacity to perform hematology and biochemistry investigations but lacks microbiology diagnostic capacity at the time of data collection. Patients have access to available diagnostic services at out-of-pocket costs despite financial hardship.

There are neither Infectious Disease Physicians nor Microbiologists to guide rational antibiotic prescribing in these hospitals. Although there were drugs and therapeutic committees in these hospitals at the time of data collection, they did not provide antimicrobial stewardship interventions such as dedicated leadership, prescriber education, and audit of prescription practices. Furthermore, there are no existing antimicrobial guidelines or formulary to leverage antibiotic prescribing practices.

### Diagnostic categories of patients prescribed antibiotics

The diagnoses of patients who were prescribed antibiotics were divided into:

- Clinical bacterial infection: when a patient develops an infection that is clinically suspected to be of bacterial origin and thus requires empiric antibiotics.
- No clinical bacterial infection/sole diagnosis of tuberculosis; these patients did not require antibiotics because the diagnosis they were being treated for was either unspecified or not a clinical bacterial infection other than tuberculosis. Therefore, it is inappropriate to prescribe antibiotics to patients in these categories. The sub-categories of this group include: 

- ‘No diagnosis’ when there was absence of a diagnosis.
- ‘Non-bacterial process with HIV’ when an HIV patient had either a non-bacterial infective process or non-infective process.
- ‘Non-infective process’ when patient had a non-communicable disease without evidence of infection
- ‘Non-bacterial infective process’ when patient had an infection which was designated as non-bacterial in origin and
- Sole diagnosis of tuberculosis.

### Definitions

The WHO AWaRe framework and the 2021 ATC Classification system was used to evaluate antibiotic use patterns as listed in the WHO Model List of Essential Medicine [15, 16].

The DDDs were calculated by multiplying the quantity field by DDD conversion factor field on the 2022 version of the ATC/DDD index using the formula DDD/100 bed days = Number of units administered in a given period (milligram) ×100/DDD (milligram)×number of days in the period×number of beds×occupancy index [16]. A bed-day was defined as overnight stay in hospital.

### Data collection and analysis

After admission to the medical or intensive care unit, patients were sequentially recruited by a nurse from each hospital who was trained in the assessment of antibiotic use. Demographic and clinical characteristics were collected at baseline using a questionnaire adapted from a previous study and encrypted in EpiCollect software (Epic, Verona WI) [6, 20]. Subsequently, the nurses review patients’ records and interview patients or ward nurses to collect information on the dose of antibiotics, their routes, and frequency of administration and duration of their use on alternate days until the end of admission. A physician reviews and verifies each completed questionnaire.

After collection, the data were extracted into Microsoft Excel, cleaned, coded, and then transferred into Stata version 16 (StataCorp LLC) for analysis. Descriptive statistics such as frequencies and percentages were used to present demographic and clinical characteristics of study participants, as well as antibiotic consumption.

## Results

### Demographic characteristics of study participants

Of 468 patients enrolled in the study, 241 (51.7%) were women. The mean age of the patients was 48.6 years (SD, 17.9). The majority were treated at MGH (245, 52.4%) (Table 1).

**Table 1:** Demographic and clinical characteristics of study participants

Parameter	Total N (%)	MH N (%)	MGH N (%)
**Overall total**	468(100)	245(52.4)	223(47.6)
**Sex**			
Female	241(51.5)	122(49.8)	119(53.4)
Male	227(48.5)	123(50.2)	104(46.6)
**Age(yrs)**			
< 25	48(10.3)	11(4.5)	37(16.6)
25–44	143(30.7)	58(24.0)	85(38.1)
45–64	171(36.8)	112(46.3)	59(26.5)
≥ 65	103(22.2)	61(25.2)	42(18.8)
Mean (SD)	48.6(17.9)	53.3(16.0)	43.5(18.6)
**Marital status**			
Single	103(22.0)	47(19.2)	56(25.1)
Married	298(63.7)	159(64.9)	139(62.3)
Separated/divorced/widowed	57(14.3)	39(15.9)	28(12.6)
**Education**			
None	197(42.1)	57(23.3)	140(62.8)
Primary	30(6.4)	18(7.3)	12(5.4)
Secondary	161(34.4)	111(45.3)	50(22.4)
Tertiary	80(17.1)	59(24.1)	21(9.4)
**Occupation**			
None	76(16.2)	37(15.1)	39(17.5)
Student	45(9.6)	19(7.8)	26(11.7)
Informal sector	215(45.9)	85(34.7)	130(54.3)
Formal sector	88(18.8)	63(25.7)	25(11.2)
Retired	44(9.4)	41(16.7)	3(1.3)
**Ward**			
Medical	431(92.1)	244(99.6)	187(83.9)
ICU	37(7.9)	1(0.4)	36(16.1)

*MH  *34 Military Hospital,  *MGH* Makeni Government Hospital  and ICU Intensive Care Unit

### Clinical diagnosis and antibiotic use

Only 180 (38.5%) patients were clinically diagnosed with a bacterial infection, of which pneumonia (80,17.1%) and gastroenteritis and other gastrointestinal infections (59, 12.6%) were the most common diagnostic categories for which antibiotics were prescribed (Table 2). Regardless of the diagnosis, 442 (94.9%) medical patients admitted either in the medical wards (405, 94.0%) or ICU (37, 100%) received at least one antibiotic. The majority (171, 95.0%) of the patients with suspected bacterial infection was prescribed an antibiotic. Of the 9 patients with no indicated diagnosis, 8(88.9%) were given an antibiotic. Nearly all patients with HIV (39, 97.5%) were prescribed an antibiotic (Table 2).

**Table 2:** Diagnostic categories of medical patients at MH and MGH

Parameter	Total participants		Received antibiotics	
	N	%	N	%
Suspected bacterial infections	180	38.5	171	95.0
Pneumonia	80	17.1	75	93.8
Gastroenteritis and other gastrointestinal infections	59	12.6	59	100.0
Skin and soft tissue infections	11	2.5	11	100.0
Sepsis with no defined focus	8	1.7	8	100.0
Urinary tract infections	7	1.5	6	85.7
Sexually transmitted and other genital infections	5	0.8	4	80.0
Central Nervous System Infections	3	0.6	3	100.0
Others: unspecified febrile illness, infective endocarditis and septic arthritis	7	1.3	5	71.4
**No clinical bacterial infections**	**286**	**60.1**	**271**	**94.8**
No diagnosis	9	1.9	8	88.9
Non-bacterial process with HIV	40	8.5	39	97.5
Non-infective process	122	26.1	116	95.1
Non-bacterial infective process	67	14.3	65	97.0
Sole diagnosis of tuberculosis	48	10.3	43	89.6
**Missing data**	**2**	**0.4**	-	-
**Hospital**				
MH	245	52.4	231	94.3
MGH	223	47.6	211	94.6
**Wards**				
Medical	431	92.1	405	94.0
ICU	37	7.9	37	100.0

*MH *34 Military Hospital and *MGH *Makeni Government Hospital, *ICU *Intensive Care Units

### Antibiotic use and consumption

Of the 813 administered antibiotic doses in the medical wards or intensive care unit (ICU) of the two hospitals, 424 (52.2%) were made in MH (Table 2). The mean number of antibiotics prescribed per patient is 1.8. Most people had two antibiotics (229, 48.9%) and the majority of the prescribed antibiotics were parenteral (685, 84.3%). The mean duration of antibiotic administration was 5 days (Table 3).

**Table 3:** Number, duration and route of antibiotic administration at MH and MGH

Parameter	N	%
**Number of antibiotics administered per patient (mean = 1.8)**		
One	172	36.8
Two	229	48.9
Three	58	12.4
Four	8	1.7
Missing	1	0.2
**Route of administration**		
Oral	128	15.7
Parenteral	685	84.3
**Duration of antibiotic use (mean duration = 5.0 days)**		
Less than 4	239	29.4
4–6	446	54.9
Greater than 6	128	15.7

The most commonly administered antibiotics were ceftriaxone (277, 34.1%), metronidazole (235, 28.9%), and levofloxacin (104, 12.8%) (Table 4). Antibiotic consumption was 23.9 DDDs per 100 bed-days for ceftriaxone, 18.0 DDDs/100 bed-days for levofloxacin, and 12.4 per 100 bed days for metronidazole (Table 5).

**Table 4:** Antibiotics and their frequency of administration in each of the study hospitals

Variable	Total drug administration	MH = 424 N (%)	MGH = 389 N (%)
Amoxicillin	2(0.2)	1(0.2)	1(0.3)
Amoxicillin-cloxacillin	28(3.4)	0(0.0)	28(7.2)
Amoxicillin-clavulanate	52(6.4)	17(4.0)	35(9.0)
Azithromycin	47(5.8)	31(7.3)	16(4.1)
Ceftriaxone	277(34.1)	135(31.8)	142(36.5)
Ciprofloxacin	41(5.1)	24(5.7)	17(4.4)
Clindamycin	1(0.1)	1(0.2)	0(0.0)
Doxycycline	2(0.2)	1(0.2)	1(0.3)
Erythromycin	2(0.2)	2(0.5)	0(0.0)
Gentamycin	22(2.7)	5(1.2)	17(4.4)
Levofloxacin	104(12.8)	104(24.5)	0(0.0)
Metronidazole	235(28.9)	103(24.3)	132(33.9)

*MH *34 Military Hospital and *MGH *Makeni Government Hospital

**Table 5:** Showing antibiotics and their frequency administration, AWaRe category and antibiotic consumption (in DDDs per 100 bed-days)

Variable	ATC code	AWaRe category	Drug administration N (%) 813(100)	DDDs/100 bed-days N (%) 66.9(100)
Amoxicillin	J01CA04	Access	2(0.2)	0.1(0.1)
Amoxicillin-cloxacillin	J01CR50	Access	28(3.4)	0.7(1.0)
Amoxicillin-clavulanate	J01CR02	Access	52(6.4)	3.9(5.8)
Azithromycin	J01FA10	Watch	47(5.8)	2.5(3.7)
Ceftriaxone	J01DD04	Watch	277(34.1)	23.9(35.7)
Ciprofloxacin	J01MA02	Watch	41(5.1)	3.5(5.2)
Clindamycin	J01FF01	Access	1(0.1)	0.1(0.1)
Doxycycline	J01AA02	Access	2(0.2)	0.1(0.1)
Erythromycin	J01FA01	Watch	2(0.2)	0.05(0.07)
Gentamycin	J01GB03	Access	22(2.7)	1.7(2.5)
Levofloxacin	J01MA12	Watch	104(12.8)	18.0(26.9)
Metronidazole	P01AB01	Access	235(28.9)	12.4(18.5)

**Fig. 1:**
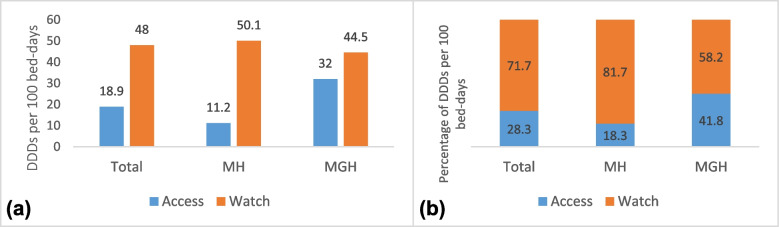
**a** Total antibiotic consumption presented as DDDs per 100 bed-days by WHO AWaRe category in two hospitals in Freetown. **b** Proportion of total antibiotic DDDs per 100 bed-days by WHO AWaRe category in the hospitals. *MH = 34 Military Hospital and MGH = Makeni Government Hospital*

The overall antibiotic consumption was 66.9 DDDs per 100 bed-days. The ACCESS antibiotics accounted for 18.9 DDDs per 100 bed-days (28.2%), whereas consumption for WATCH antibiotics was 48.0 DDDs per 100 bed-days (71.7%). No RESERVE antibiotics were prescribed. The antibiotic consumption was lower in MH (61.3 DDDs per 100 bed-days) than MGH (76.5 DDDs per 100 bed-days), but the consumption of the WATCH antibiotics was higher in MH (81.7%) than MGH (58.2 (Fig. 1)).

## Discussion

We used the 2021 ATC/DDD and the WHO AWaRe classifications to determine the prevalence of antibiotic use, and assess antibiotic consumption among patients admitted to the medical wards of two hospitals in Sierra Leone [15, 16].

About 95% of medical patients admitted to both hospitals were prescribed antibiotics, consuming 66.9 DDDs of antibiotics per 100 bed-days. Previous studies in Sierra Leone reported similar antibiotic prescription rates for hospitalized patients (82%) and in surgery (92%) [6, 22]. In Nigeria and Ethiopia, similarly high levels of antibiotic use were also reported [23, 24]. This pattern of prescription of antibiotics across Africa indicate similar challenges with rational antibiotic prescribing and call for greater coordination among African governments in AMR prevention and control.

Only 38.5% of the reasons for prescribing antibiotics were suspected bacterial diseases. Whether this presumptive diagnosis represents a true bacterial infection is a question that could be answered in future studies. Most importantly, many patients with non-bacterial diseases or without a documented diagnosis have been inappropriately prescribed an antibiotic, similar to studies in Japan and India [25, 26]. Antibiotic prescription should be guided by laboratory support to prevent inappropriate use, especially to treat non-bacterial diseases. However, in many hospitals in Sierra Leone, the laboratory capacity for microbiological methods is limited [6]. One plausible strategy to address this problem is the use of point-of-care tests to quickly distinguish bacteria from non-bacterial diseases on the bed side [27]. Biomarkers such as procalcitonin can be useful diagnostic tools as elevated levels have been associated with febrile bacterial infections but not with non-bacterial febrile illness [28].

Similar to previous studies [6, 22], the most commonly used antibiotic was ceftriaxone, followed by metronidazole and levofloxacin. The high use of ceftriaxone may explain the increased rates of extended-spectrum β-lactamase-producing Gram-negative bacilli reported in some hospitals in Sierra Leone [17, 19, 29]. Similarly, the high rates of use of levofloxacin in one of the hospitals may have contributed to resistance, which may potentially affect Sierra Leone’s national efforts to prevent and control multi-drug resistance tuberculosis [30, 31]. Thus, the need to explore different stewardship interventions in our hospitals and different techniques to evaluate appropriate antibiotic prescribing practices [32, 33].

WHO recommends that the narrow-spectrum ACCESS antibiotics should be used more frequently to reduce the selection pressure associated with the broad-spectrum WATCH and RESERVE antibiotics [34]. In our study, the estimated consumption of the ACCESS antibiotics was lower than the WATCH antibiotics (28.2% vs. 71.7%) in contrast with the WHO recommendation of having at least 60% consumption of ACCESS antibiotics [34], reinforcing the need for different AMS strategies such as de-escalation and restriction of antibiotics, and training of healthcare workers. None of the patients was prescribed a RESERVE antibiotic similar to data reported in a previous study in outpatient settings of the national referral hospitals in Sierra Leone [35]. Although it is more or less considered good stewardship practices not to use RESERVE antibiotics, it should be possible to use RESERVE antibiotics in the care of critically ill patients.

Our study has strengths and limitations. Although the findings of this study are not nationally representative, it is the first study in Sierra Leone to describe antibiotic use patterns and consumption among medical patients at two hospitals in different geographic regions. Hence, its evidence has important policy implications for antibiotic stewardship programs. We did not clinically verify the diagnosis of bacterial and non-bacterial diseases made by the clinicians at the bedside. Therefore, our findings may not represent the true picture of the common diagnoses that lead to antibiotic prescriptions in these hospitals.

## Conclusion

Our study showed a high prevalence of antibiotic prescribing in two hospitals in different geographic regions of Sierra Leone. Antibiotic consumption was highest with ceftriaxone, followed by levofloxacin and metronidazole. Given the high rate of consumption of antibiotics in the WATCH category of the AWaRe classification, there is a need to initiate surveillance of antibiotic consumption and establish facility-based antibiotic stewardship in these hospital settings.

## Data Availability

The data supporting this article is available in the repository of University of Sierra Leone and will be made easily available on request to the corresponding author when required.

## Abbreviations

AMR: Antimicrobial Resistance
AMS: Antimicrobial Stewardship
ATC: Anatomical Chemical and Therapeutics
AWaRe: Access Watch and Reserve
COVID-19: Coronavirus disease 2019
DDD: Defined Daily Dose
LMIC: Low- and middle-income countries
SORT: Structured Operational Research and Training Initiative
TDR: The Special Program for Research and Training in Tropical Diseases
WHO: World Health Organization

## References

[1] O’Neill J. Tackling drug-resistant infections globally: final report and recommendations. London: Review on Antimicrobial Resistance, 2016. Available at: https://amr-review.org/sites/default/files/160518_Final%20paper_with%20cover.pdf. Accessed 4 Mar 2022.

[2] Antimicrobial Resistance Collaborators (2022). Global burden of bacterial antimicrobial resistance in 2019: a systematic analysis. Lancet.

[3] Aldeyab MA, Kearney MP, McElnay JC, Magee FA, Conlon G, MacIntyre J (2012). A point prevalence survey of antibiotic use in four acute-care teaching hospitals utilizing the european surveillance of antimicrobial consumption (ESAC) audit tool. Epidemiol Infect.

[4] Bell BG, Schellevis F, Stobberingh E, Goossens H, Pringle M (2014). A systematic review and meta-analysis of the effects of antibiotic consumption on antibiotic resistance. BMC Infect Dis.

[5] Selcuk A (2021). The point prevalence and inappropriateness of antibiotic use at hospitals in Turkey: a systematic review and meta-analysis. J Chemother.

[6] Lakoh S, Adekanmbi O, Jiba DF, Deen GF, Gashau W, Sevalie S, Klein EY (2020). Antibiotic use among hospitalized adult patients in a setting with limited laboratory infrastructure in Freetown Sierra Leone, 2017–2018. Int J Infect Dis.

[7] Klein EY, Van Boeckel TP, Martinez EM, Pant S, Gandra S, Levin SA, Goossens H, Laxminarayan R (2018). Global increase and geographic convergence in antibiotic consumption between 2000 and 2015. Proc Natl Acad Sci U S A.

[8] Cox JA, Vlieghe E, Mendelson M, Wertheim H, Ndegwa L, Villegas MV, Gould I, Levy Hara G (2017). Antibiotic stewardship in low- and middle-income countries: the same but different?. Clin Microbiol Infect.

[9] Pierce J, Apisarnthanarak A, Schellack N, Cornistein W, Maani AA, Adnan S, Stevens MP (2020). Global antimicrobial stewardship with a focus on low- and middle-income countries. Int J Infect Dis.

[10] Kakkar AK, Shafiq N, Singh G, Ray P, Gautam V, Agarwal R, Muralidharan J, Arora P (2020). Antimicrobial Stewardship Programs in Resource constrained environments: understanding and addressing the need of the Systems. Front Public Health.

[11] Rodríguez-Baño J, Rossolini GM, Schultsz C, Tacconelli E, Murthy S, Ohmagari N, Holmes A, Bachmann T, Goossens H, Canton R, Roberts AP, Henriques-Normark B, Clancy CJ, Huttner B, Fagerstedt P, Lahiri S, Kaushic C, Hoffman SJ, Warren M, Zoubiane G, Essack S, Laxminarayan R, Plant L (2021). Key considerations on the potential impacts of the COVID-19 pandemic on antimicrobial resistance research and surveillance. Trans R Soc Trop Med Hyg.

[12] Hirabayashi A, Kajihara T, Yahara K, Shibayama K, Sugai M (2021). Impact of the COVID-19 pandemic on the surveillance of antimicrobial resistance. J Hosp Infect.

[13] Yin J, Li H, Sun Q (2021). Analysis of antibiotic consumption by AWaRe classification in Shandong Province, China, 2012–2019: a Panel Data Analysis. Front Pharmacol.

[14] WHO access, watch, reserve, classification of antibiotics for evaluation and monitoring of use. 2021 ‎AWaRe classification‎. Available from: https://www.who.int/publications/i/item/2021-aware-classification. Accessed 3 Dec 2021.

[15] Guidelines for ATC classification and DDD assignment 2021. Available at: https://www.whocc.no/filearchive/publications/2021_guidelines_web.pdf Accessed 2 Dec 2021.

[16] WHO. *World Health Organization Model List of Essential Medicines, 22nd List* World Health Organization; Geneva, Switzerland: 2021. Available at: https://apps.who.int/iris/bitstream/handle/10665/345533/WHO-MHP-HPS-EML-2021.02-eng.pdf. Accessed 2 Dec 2021.

[17] Lakoh S, Li L, Sevalie S, Guo X, Adekanmbi O, Yang G, Adebayo O, Yi L, Coker JM, Wang S, Wang T, Sun W, Habib AG, Klein EY (2020). Antibiotic resistance in patients with clinical features of healthcare-associated infections in an urban tertiary hospital in Sierra Leone: a cross-sectional study. Antimicrob Resist Infect Control.

[18] Lakoh S, Firima E, Williams CEE, Conteh SK, Jalloh MB, Sheku MG, Adekanmbi O, Sevalie S, Kamara SA, Kamara MAS, Barrie U, Kamara GN, Yi L, Guo X, Haffner C, Kamara MN, Jiba DF, Namanaga ES, Maruta A, Kallon C, Kanu JS, Deen GF, Samai M, Okeibunor JC, Russell JBW (2021). An intra-COVID-19 assessment of hand hygiene facility, policy and staff compliance in two hospitals in Sierra Leone: is there a difference between Regional and capital city hospitals?. Trop Med Infect Disease.

[19] Lakoh S, Yi L, Sevalie S, Guo X, Adekanmbi O, Smalle IO, Williams N, Barrie U, Koroma C, Zhao Y, Kamara MN, Cummings-John C, Jiba DF, Namanaga ES, Deen B, Zhang J, Maruta A, Kallon C, Liu P, Wurie HR, Kanu JS, Deen GF, Samai M, Sahr F, Firima E (2022). Incidence and risk factors of surgical site infections and related antibiotic resistance in Freetown, Sierra Leone: a prospective cohort study. Antimicrob Resist Infect Control.

[20] Cole CP, Routledge P (2018). An evaluation of rational prescribing in hospital outpatient practice in Sierra Leone and assessment of affordability of a prescription as an outcome. Pan Afr Med J.

[21] Kanu JS, Khogali M, Hann K, Tao W, Barlatt S, Komeh J, Johnson J, Sesay M, Vandi MA, Tweya H, Timire C, Abiri OT, Thomas F, Sankoh-Hughes A, Molleh B, Maruta A, Harries AD (2021). National antibiotic consumption for human use in Sierra Leone (2017–2019): a cross-sectional study. Trop Med Infect Dis.

[22] Lakoh S, Kanu JS, Conteh SK, Russell JBW, Sevalie S, Williams CEE, Barrie U, Kabia AK, Conteh F, Jalloh MB, Deen GF, Kabba MS, Lebbie A, Kamara IF, Fofanah BD, Maruta A, Kallon C, Sahr F, Samai M, Adekanmbi O, Yi L, Guo X, Kamara RZ, Jiba DF, Okeibunor JC, Yendewa GA, Firima E. High levels of surgical antibiotic prophylaxis: implications for hospital-based antibiotic stewardship in Sierra Leone. Antimicrob Steward Healthc Epidemiol. 2022;2(1):e111. 10.1017/ash.2022.252. PMID: 36483422; PMCID: PMC9726495. 10.1017/ash.2022.252 PMC9726495 36483422

[23] Abubakar U (2020). Antibiotic use among hospitalized patients in northern Nigeria: a multicenter point-prevalence survey. BMC Infect Dis.

[24] Ayele Y, Taye H (2018). Antibiotic utilization pattern for surgical site infection prophylaxis at Dil Chora referral hospital surgical ward, dire Dawa, Eastern Ethiopia. BMC Res Notes.

[25] Kimura Y, Fukuda H, Hayakawa K, Ide S, Ota M, Saito S, Ishikane M, Kusama Y, Matsunaga N, Ohmagari N (2019). Longitudinal trends of and factors associated with inappropriate antibiotic prescribing for non-bacterial acute respiratory tract infection in Japan: a retrospective claims database study, 2012–2017. PLoS ONE.

[26] A prospective study on the antimicrobial usage. In the medicine department of a tertiary care teaching hospital. J Clin Diagnostic Res. 2013;7:1343–6. 10.7860/JCDR/2013/6265.3125. 10.7860/JCDR/2013/6265.3125 PMC3749632 23998062

[27] Abedini A, Kiani A, Emami H, Touhidi MH (2019). Serum procalcitonin level as a predictor of bacterial infection in patients with COPD exacerbation. Tanaffos.

[28] Schuetz P, Beishuizen A, Broyles M, Ferrer R, Gavazzi G, Gluck EH, Del González J, Jensen JU, Kanizsai PL, Kwa ALH, Krueger S, Luyt CE, Oppert M, Plebani M, Shlyapnikov SA, Toccafondi G, Townsend J, Welte T, Saeed K (2019). Procalcitonin (PCT)-guided antibiotic stewardship: an international experts consensus on optimized clinical use. Clin Chem Lab Med.

[29] Lakoh S, Yi L, Russell JBW, Zhang J, Sevalie S, Zhao Y, Kanu JS, Liu P, Conteh SK, Williams CEE, Barrie U, Sheku MG, Jalloh MB, Adekanmbi O, Jiba DF, Kamara MN, Deen GF, Okeibunor JC, Yendewa GA, Guo X, Firima E (2022). The burden of surgical site infections and related antibiotic resistance in two geographic regions of Sierra Leone: a prospective study. Ther Adv Infect Dis.

[30] Leski TA, Stockelman MG, Bangura U, Chae D, Ansumana R, Stenger DA, Vora GJ, Taitt CR (2016). Prevalence of quinolone resistance in enterobacteriaceae from Sierra Leone and the detection of qnrB Pseudogenes and modified LexA binding Sites. Antimicrob Agents Chemother.

[31] Lakoh S, Yendewa GA (2022). Multidrug-resistant tuberculosis in Sierra Leone. Lancet Glob Health.

[32] Mendelson M, Morris AM, Thursky K, Pulcini C (2020). How to start an antimicrobial stewardship programme in a hospital. Clin Microbiol Infect.

[33] Lakoh S, Bawoh M, Lewis H, Jalloh I, Thomas C, Barlatt S, Jalloh A, Deen GF, Russell JBW, Kabba MS, Batema MNP, Borgstein C, Sesay N, Sesay D, Nagi NK, Firima E, Thomas S (2023). Establishing an antimicrobial stewardship program in Sierra Leone: a report of the experience of a low-income country in West Africa. Antibiot (Basel).

[34] Sharland M, Gandra S, Huttner B, Moja L, Pulcini C, Zeng M, Mendelson M, Cappello B, Cooke G, Magrini N, EML Expert Committee and Antibiotic Working Group.; (2019). Encouraging AWaRe-ness and discouraging inappropriate antibiotic use-the new 2019 Essential Medicines List becomes a global antibiotic stewardship tool. Lancet Infect Dis.

[35] Lakoh S, John-Cole V, Luke RDC, Bell N, Russell JBW, Mustapha A, Barrie U, Abiri OT, Coker JM, Kamara MN, Coker FJ, Adekanmbi O, Kamara IF, Fofanah BD, Jiba DF, Adeniji AO, Kenneh S, Deen GF, Moon TD, Yendewa GA, Firima E (2023). Antibiotic use and consumption in Freetown, Sierra Leone: a baseline report of prescription stewardship in outpatient clinics of three tertiary hospitals. IJID Reg.

